# Supplementary material for: Research on the evolution mechanism of main control factors for coalbed methane extraction
Source: PLoS One. 2026 Jan 2;21(1):e0338859. doi: 10.1371/journal.pone.0338859 (PMC12758805; doi:10.1371/journal.pone.0338859)
Supplement: Attachment — Submitted filename: Comments.docx [file pone.0338859.s001.docx]

**Comments**

1. [Page 1, Line 8]: Take into consideration defining "self-developed experimental device" and briefly discuss any novelty or characteristics.
2. [Page 1, Line 14]: There is no specific reference to back up the fitting advantage of DR in the phrase comparing it to the Langmuir and BET models; think about citing pertinent research.
3. The expression "gas components stored in the form of micropore filling" might be more clearly articulated as "gas molecules are primarily adsorbed in micropores."
4. [Page 2, Line 6]: The term "extremely complex" about gas migration in coal lacks precision. It would be beneficial to delineate the various forms of complexity, such as heterogeneity and dual porosity, among others. Particles are primarily retained within the confines of micropores.
5. 6. [Page 2, Line 14]: Reference [10] asserts that "bright coal had poor pore connectivity." Please provide a more detailed technical explanation or specify the parameters related to "poor connectivity."
6. 7. [Page 2, Line 23]: The sentence beginning with "Numerous studies..." lacks specificity. It would be beneficial to articulate a particular knowledge gap that your research aims to fill.
7. [Page 3, Line 1]: Specify the precise mass of the coal sample utilized in each experiment to enhance reproducibility.
8. [Page 3, Line 6]: The term "appropriate number of swabs" lacks precision; it would be beneficial to quantify this or clarify the intended purpose of the swabs.
9. [Page 3, Line 12]: It would be beneficial to specify the time required to achieve adsorption equilibrium at each pressure point.
10. [Page 4, Line 3]: In the discussion of Figure 2, where it states that the “adsorbed gas volume increased sharply,” please include specific time or pressure values to substantiate this observation.
11. [Page 4, Line 20]: The formatting of equation numbers (1), (2), and (3) lacks consistency and proper alignment.
12. [Page 4, Line 28]: The phrase “still 0.9765” could be revised for a more formal tone to "remained relatively high at 0.9765."
13. [Page 5, Line 5]: It would be beneficial to compare your desorption result (14.7 ml/g) with values from existing literature, as this could aid in validating the experiment.
14. [Page 5, Line 9]: The statement "some gas escaped..." could be enhanced by incorporating additional detail or quantifying the error associated with gas loss.
15. [Page 6, Line 2]: Kindly furnish the boundary conditions or geometric details employed in the COMSOL simulations.
16. [Page 6, Line 18]: Please provide clarification on the criteria used to assess fitting quality; was it determined through R², RMSE, or visual overlap?
17. [Page 7, Line 7]: There is an inconsistency in the model naming; it alternates between “single-hole” and “single-pore.” Kindly ensure that the terminology is consistent throughout the document.
18. [Page 7, Line 22]: The assertion "struggled to accurately predict... accurately" is qualitative. It would be beneficial to include numerical fitting error values for support.
19. [Page 8, Line 3]: The conclusion would be strengthened by including a concise statement regarding the practical applications of the findings in coalbed methane extraction operations.
20. [Page 8, Line 10]: It may be beneficial to highlight the innovative aspects of the study in the conclusion. What distinguishes this work from previous research?
