# Supplementary material for: Research on the evolution mechanism of main control factors for coalbed methane extraction
Source: PLoS One. 2026 Jan 2;21(1):e0338859. doi: 10.1371/journal.pone.0338859 (PMC12758805; doi:10.1371/journal.pone.0338859)
Supplement: Attachment — Submitted filename: Reply_to_the_review_comments_auresp_2.docx [file pone.0338859.s004.docx]

# Reply to the review comments

Reviewer #3: This manuscript has been systematically revised in response to the previous review comments, with a significant improvement in overall quality and a notable enhancement in the scientific rigor and completeness of the research. However, combined with the content of the revised version, there are still some minor details that need further clarification and improvement, as follows:

1. In Step 3 of Section 1.4, the specific values or range of the "specified pressure" have not been stated.

Thank you to the reviewers for their careful reading. It was indeed not specified what the pressure conditions were. Now it has been revised and highlighted in the text.

3. The reference gas was charged into the reference vessel at a pressure of 1–5 MPa. The valve between the reference and coal-sample vessels was then opened to initiate the gas-adsorption process.

2. The three subfigures in Figure 5 can be distinguished by labeling them as (a), (b), and (c).

Thank you to the reviewers for your careful reading. We have made revisions according to your suggestions.

3. The following papers may be useful: https://doi.org/10.1016/j.ijmst.2022.11.002; DOI: 10.1016/j.jngse.2015.01.008; DOI: 10.1080/15567036.2021.1936692; https://doi.org/10.1016/j.energy.2022.124228

Thank the reviewers for their valuable suggestions; the recommended references are extremely valuable and provide important support for the research on gas.

4. In the main text of Section 3.2, the terms "single-pore model" and "dual-pore model" are used, while the legends in Figures 7 and 8 are labeled as "Single-hole model fitting" and "Two-hole model fitting". It is suggested to unify the terminology to ensure consistency throughout the manuscript.

Thank you to the reviewers for your careful reading. We have made revisions according to your suggestions.
